# Non-local mind from the perspective of social cognition

**DOI:** 10.3389/fnhum.2013.00107

**Published:** 2013-04-02

**Authors:** Jonas Chatel-Goldman, Jean-Luc Schwartz, Christian Jutten, Marco Congedo

**Affiliations:** Gipsa-lab, UMR 5216 CNRS, Grenoble INP, Université Joseph Fourier, Université StendhalGrenoble, France

**Keywords:** hyperscanning, social interaction, brain and body coupling, empathy, joint action, theory of mind

## Abstract

Two main conceptual approaches have been employed to study the mechanisms of social cognition, whether one considers isolated or interacting minds. Using neuro-imaging of subjects in isolation, the former approach has provided knowledge on the neural underpinning of a variety of social processes. However, it has been argued that considering one brain alone cannot account for all mechanisms subtending online social interaction. This challenge has been tackled recently by using neuro-imaging of multiple interacting subjects in more ecological settings. The present short review aims at offering a comprehensive view on various advances done in the last decade. We provide a taxonomy of existing research in neuroscience of social interaction, situating them in the frame of general organization principles of social cognition. Finally, we discuss the putative enabling role of emerging non-local social mechanisms—such as interpersonal brain and body coupling—in processes underlying our ability to create a shared world.

## Introduction

Recent years have seen a flourishing interest in exploring underlying mechanisms of social interaction, as illustrated by a recent special topic in this journal. Motivated by the study of multiple interacting individuals in ecological social contexts (Hari and Kujala, 2009; Schilbach, 2010; Dumas, 2011) this research trend departs from traditional focus on sole investigation of brains in isolation (see Table 1). A central question here is to what extent cognition is shaped—or even constituted (De Jaegher et al., 2010)—by mutual interplay and co-regulated coupling between interacting agents embedded in their environment (Coey et al., 2012; Hasson et al., 2012; Krueger and Michael, 2012). First results in this direction come from sparse and heterogeneous studies, with experimental paradigms ranging, e.g., from use of economic games (De Vico Fallani et al., 2010), music playing or singing (Müller and Lindenberger, 2011), hand movement imitation (Dumas et al., 2010), speech production, and perception (Stephens et al., 2010), to facial communication of affect (Anders et al., 2011).

**Table 1:** **Comparison between paradigms of isolation and interaction in studies on social cognition**.

	**Isolated approach**	**Interactive approach**
Investigation methods	Neuro-imaging studies implying subjects in isolation.	Neuro-imaging studies with subjects engaged in interaction.
Experimental paradigms	Observational scenarios (offline).	Interacting and more ecological scenarios (online).
Some characteristics	Mature concepts and theories.	Recent and growing theoretical framework.
	Well-known and clear experimental paradigms.	Studies in ecological settings, harder to set up.
	Existing work includes studies on impaired population as well as developmental and comparative studies.	No work to date either on impaired population, or on developmental or comparative studies.
Benefits	Enable to give ground knowledge on neural underpinnings of a variety of social processes.	Only way to investigate the dynamics of social processes involved during mutual interplay.
		Social brain processes at work may be different during online reciprocal interaction.
Prime importance in learning	Yes	Yes
Explanatory strategies	First- and third-person accounts of social cognition, modular and individualistic explanations, internalized processes.	Second-person account of social cognition, enactive perspective, dynamical concepts: synergies, metastability, coordination, etc.
Theories	Theory-theory, simulation-theory, etc.	Strong/moderate interactionism, interactive brain hypothesis, non-local correlations, etc.

Since a large conceptual gap remains between the “isolated” and “interactive” approaches (Di Paolo and De Jaegher, 2012; Konvalinka and Roepstorff, 2012), much effort is needed today to situate new contribution in the complex picture of social cognition. It was claimed recently that social cognition itself may be fundamentally different from an interactor's vs. from an observer's point of view (Schilbach et al., in press). In consequence, when entering the multiple- brain and body methodological framework we need to disentangle the social mechanisms revealed in isolation paradigms (offline) from those that are presumed proper to genuine interaction (online).

**Figure 1:**
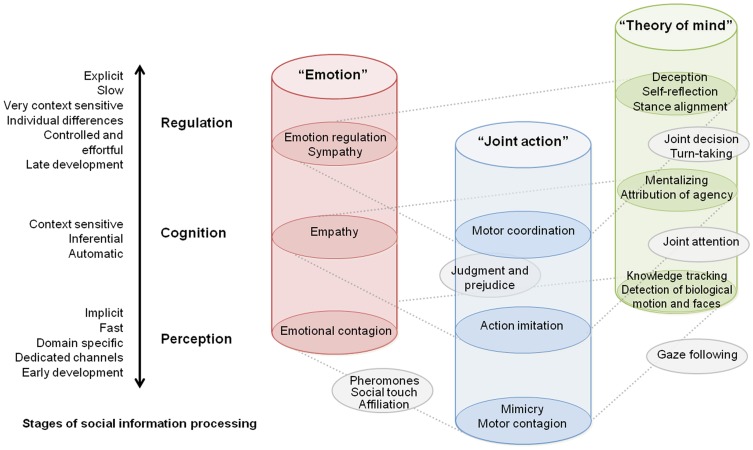
**Taxonomy of current studies on interacting brain and bodies presented from the perspective of investigated social processes.** Each cylinder represents a distinct research cluster adopted by the community. The schematic view describes how social neuroscience research aggregated on three main categories depending on investigated social cognitive processes. Vertical dimension of the diagram situates these studies in the context of general organization principles of social cognition (see main text). This diagram should not be seen as architecture of neural mechanisms *per se*, but as a general map of social processes as they were inquired in actual studies.

In this paper, we aim at facilitating the conceptualization when investigating cognitive processes (Box 1) during social interaction. Using a reductionist approach, we propose a classification of explored functions into distinct domains and stages of information processing (Figure 1).

Box 1 **Operational definitions.**

- Cognitive process
  High-level function or task, at least in part reducible to a sequence of operations (brain mechanisms).
- Mechanism
  Operation on information content.
- Non-local mechanism
  Dependent operations between two or more brains that operate at least in part on shared information content.
- Hyperscanning
  Simultaneous collection of brain activity from two or more (interacting) subjects.
- Dual-(EEG/fMRI)
  Hyperscanning-(EEG/fMRI) on two (interacting) subjects.

This comprehensive frame:

- Enables to fit the recent and heterogeneous advances made in research on interacting individuals into the bigger picture of social cognition.
- Highlights a categorization of current works into *three distinct groups*, each corresponding to the use of specific experimental methodologies, types of interaction and theoretical approaches.
- Uncovers the domains and processes of social cognition for which we still lack a fine understanding of interactive mechanisms, i.e., mechanisms that have not been explored yet, or for which new methodologies may be applied.

In the following, we give clarifications on the different dimensions of the drawing and their implications in terms of methodological as well as conceptual approaches. Then we consider a few examples of studies on human interaction as an illustration for the provided taxonomy. We conclude discussing the potential enabling role of emergent non-local mechanisms on social processes.

## Horizontal dimension: domains of investigated social processes

Each cylinder in Figure 1 represents a research focus adopted by the community. Up to now three main clusters gather most of the neuro-imaging studies in neuroscience of social interaction, whether focusing on the general themes of theory of mind (ToM), emotions in a social context, or joint action. Few outer studies also begin to link up these different categories of explored social processes. Interestingly, this domain-based distinction corresponds at least in part to specific brain mechanisms. It is admitted, for instance that empathy and mind reading rely on different neuronal circuitry and display different ontogenetic and phylogenetic trajectories (Singer, 2006). Meanwhile, some brain structures are known to play a critical role in multiple aspects of social cognition. Finally, several investigations demonstrate an interaction across processes depicted in Figure 1. For example, mimicry can contribute to an empathic response (Singer and Lamm, 2009), and motor contagion arises from the observation of biological movements and could in turn be a first step for automatic inference of goal-directed actions (Blakemore and Frith, 2005).

## Vertical dimension: stages of social information processing

Besides the aforementioned partitioning, we propose to situate neuroimaging studies of social interaction within commonly recognized organization levels of social cognition. To do so, we situate the social processes along bipolar continua together with their key attributes often considered in the literature. Extensive source material in line with this architecture can be found e.g., in Adolphs (2010), highlighting multiple stages of social information processing and in Frith and Frith (2008), showing the importance of implicit vs. explicit processes of social cognition. Progressing from lower to higher stages of social information processing, from perception through cognition to regulation, in Figure 1 we highlight the changes in attributes, such as automaticity and control, process speed, sensitivity to context, age of development in normal infant, and probably phylogenetic trajectory.

## A categorization in terms of investigated mechanisms, experimental paradigms, and theoretical standpoint

An essential aspect when investigating neural mechanisms at play during social interaction consists in analyzing brain activity in the light of behavioral data, ideally spanning the whole dimension of socialness. In this regard, works in the three clusters have used different strategies. Most studies on ToM during interaction have employed experimental paradigms inspired from game theory, with the aim of studying what a subject infers about the mental state of the other. While this framework offers elegant mathematical formalizations (for a review see Lee, 2008), it generally fails to recreate the dynamics of real-life interplay due to the use of turn-based, non-ecological scenarios. On the other hand, studies on emotions between interacting participants are challenged by the absence of objective measures of affect. Researchers have tried to circumvent this problem with the use of subjective empathy test or by adopting pseudo-interactive approaches inspired from information theory, where empathy is reduced to a quantifiable transmission of emotion from a sender to a receiver. Finally, works on joint action have benefited from fine physical measures of interpersonal synchrony captured at the motor level in more ecological settings. We argue that the necessary use of different data collection strategies has narrowed down the experimental paradigms for each type of social interaction: turn-based for works on ToM, unidirectional for research on affect, and reciprocal online interaction for works on joint action. While real-life social interactions are most of the time unconstrained and co-regulated, it is of utmost importance to revisit experimental paradigms—especially those used when investigating ToM and affect—introducing a truly interactive and engaged perspective (Hari and Kujala, 2009; Schilbach et al., in press).

Interestingly, conceptual approaches differ from one research cluster to the other, whereas they are quite similar within each cluster. This further categorization from a theoretical standpoint is not unexpected since scholars tend to share the prevalent theories in their specific research area. Accordingly, works around ToM are deeply entrenched in a cognitivist, third-person view on the individual as a passive recipient of information (e.g., King-Casas et al., 2005; De Vico Fallani et al., 2010). Works on emotions are mostly related to a simulationist and embodied account of social cognition (e.g., Anders et al., 2011; Babiloni et al., 2012). Finally research on joint action is generally accompanied by an interactive and dynamical view of the foundations of our social abilities (e.g., Lindenberger et al., 2009; Dumas et al., 2010). The latter conceptual trend has gained momentum recently and begins to spread to research on mind-reading through an expanding literature in the fields of joint and shared attention, largely inspired from developmental psychology. Integration of these conflicting theoretical frameworks and mapping to underlying intra- and inter-brain mechanisms is one of the future challenges of social neurosciences.

## Examples from the “ToM” study ensemble

We now consider some example studies in each of the aforementioned research cluster. In line with a cognitivist view of the mind, a number of hyperscanning-fMRI studies in the “ToM” research ensemble have solely focused on cognitive processes involved during cooperative, trust and/or economic games. These experiments identified key neural mechanisms in the social domain: reputation building and reciprocity engage caudate nucleus (King-Casas et al., 2005), assignment of credit and social agency arrange in spatial patterns along the cingulate cortex (Tomlin et al., 2006), and human cooperation modulates activity in the caudate and putamen reward centers (Krill and Platek, 2012). However, here the systematic use of turn-based and pseudo-interactive paradigms in addition to a limited analysis of inter-brain connectivity did not take full advantage of the hyperscanning methodological approach.

While still keeping a turn-based constraint on social interaction, a dual-EEG study (De Vico Fallani et al., 2010) investigated on a millisecond timescale how cooperative or defective behavior changes the functional organization at both intra and inter-individual levels. Authors developed new tools adapted from graph theory enabling to obtain a connectivity pattern—devised “hyper-brain network”- that represents both information flows among the cortical regions within single brain as well as the relations among the areas of two distinct brains. Hyper-brain networks were then compared between different strategies adopted by the subjects during an Iterative Prisoner's Dilemma game. Interestingly, two-defector couples showed significantly less inter-brain links and increased tendency to form two separate subgraphs than couples playing cooperative or tit-for-tat strategies, and decision to defect could be predicted from changes in connectivity patterns in the hyper-brain networks.

Using similar analysis framework to explore the interpersonal dynamics of mentalizing during a four-person card game, Astolfi et al. (2010) found that only the players belonging to the same team showed significant functional connectivity in alpha, beta and gamma bands. They also found a causal relation between brain signals estimated in the prefrontal area of the team's leader and anterior cingulate cortex (ACC) of his partner. Finally, with an innovative dual-NIRS setup, Cui et al. (2012) reported increased coherence in superior frontal cortices during cooperation as compared to competition, which was associated with an increase in cooperation performance during the game and could not be revealed by single-brain analysis alone.

These works demonstrate that simultaneous data collection (hyperscanning) and innovative joint analysis tools make possible to characterize brain activity at both an individual and joint (non-local) level.

## Examples from the “joint action” study ensemble

Using EEG-hyperscanning, Tognoli et al. (2007) revealed an oscillatory component over the right centroparietal regions in the mu (9–12 Hz) range, which proved to be sensitive to elementary forms of spontaneous social coordination. Naeem et al. (2012) replicated this study and found similar results with intentional movement coordination. However, none of these works examined directly the role of interbrain synchronization in coordinated action.

In another EEG-hyperscanning experiment, Dumas et al. (2010) explored the functional dynamics of action imitation and coordination during free hand movements. This ecological task allowed for a moment-to-moment free interaction, while keeping a structured experiment through the use of dual video recording with which instants of behavioral synchrony were precisely segregated. By means of functional connectivity and surrogate data the authors demonstrated the emergence of interbrain neural synchronizations among alpha-mu, beta, and gamma bands in the centroparietal regions of the two interacting partners. Asymmetrical pattern were found, and interpreted as modulation reflecting the differential roles of model and imitator. Importantly, the absence of significant difference between imitative and non-imitative episodes showed that interbrain synchronizations did not reflect the execution and perception of similar movements exclusively.

When investigating action coordination, it is essential to cast away interbrain synchronization that merely reflects similar neural responses to the shared sensory inputs and motor outputs. This problem came up, for instance, in another ecological study of Lindenberger et al. (2009), which observed an increase of phase synchronization in the theta band within and between the brains in pairs of guitarists while they played a melody together. However, as the authors discuss, interbrain synchronizations might have arisen (at least partially) because subjects movements were externally synchronized by a metronome.

Finally, adopting a different approach to study interpersonal coordination Dodel et al. (2011) explored signature of team performance during simulated combat. They proposed that EEG activity of the team members evolves along a particular manifold, the geometry of which would reflect task related constraints as well as effects of team coordination. They found that expertise affects dimensionality of this manifold, a result in line with recent accounts on interpersonal synergies (Riley et al., 2011).

## Examples from the “emotion” study ensemble

To the best of our knowledge, the dynamical and reciprocal aspects of interpersonal affective mechanisms have never been explored in a hyperscanning setting. The reasons may stem in the absence of objective marker of affective processing, in the difficulty to design ecological experiments or in the numerous modulatory factors that might drastically impact on empathic brain response (see De Vignemont and Singer, 2006). A first step in this direction was taken by Anders et al. (2011), who investigated facial communication of affects between romantic partners in a differed fMRI study. Subjects were assigned the roles of sender and receiver of the affective information, hence the moment-to-moment mutual adaption intrinsic to a closed and undisrupted perception-action loop could not be explored. Yet analysis provided evidence for emotion-specific information encoded in similar distributed anterior temporal, insular and somato-motor regions in the sender's and perceiver's brain, a result that was interpreted as supporting theories on embodied simulation.

Collection of autonomic data in addition to brain data may provide a better understanding of neural underpinnings of affective processes. While this has been put forward for investigations on single individuals (Sequeira et al., 2009) quantification of social effects on human physiology may be even more important in the exploration of affective mechanisms at an inter-individual level. Recent evidence show that during social interaction interpersonal coupling not only occurs at brain or behavioral level (see e.g., Oullier et al., 2008; Krueger and Michael, 2012) but also at a more general physiological level. In a field observation carried out during a collective fire-walking ritual, Konvalinka et al. (2011) identified synchrony over time of heart rate dynamics between active participants with their related onlookers, but not unrelated observers. In another ecological study, Müller and Lindenberger (2011) observed oscillatory couplings of cardiac and respiratory activity among singers and conductor engaged in choir singing. Using effective connectivity measures they highlighted causal effects of the conductor on the singers at high modulation frequencies, and dissociated the different voices of the choir using network analysis based on graph theory. In shedding light on such socially modulated synchronizations of the autonomic systems, these works demonstrate that opening an additional window on our concealed physiological states is unavoidable to draw a complete picture of affective mechanisms in place during social interaction. The latter example also suggests that music playing in ensemble might represent a naturalistic methodological approach for the investigation of inter-individual affective and coordinative processes. Its practicability for neuroimaging was demonstrated in a recent hyperscanning-EEG experiment (Babiloni et al., 2011).

## Toward a holistic view of various mechanisms at play during complex social interaction

Finally, we review some studies that seem relevant as they begin to bridge the gaps between the three clusters of Figure 1. Capitalizing on the idea that joint attention may be best captured in online interactions (Wilms et al., 2010; Schilbach et al., in press), two recent studies have investigated its neural underpinnings through dual-fMRI (Saito et al., 2010) and dual-EEG (Lachat et al., 2012). The former observed that paired subjects showed higher correlations than non-paired subjects in the right inferior frontal gyrus—a region part of the mirror neuron system—when following partner's gaze. In the latter, alpha and mu oscillatory activities over centro-parieto-occipital scalp regions were demonstrated to be electrophysiological correlates of joint attention. Likewise, using gaze-contingent stimuli during truly interactive paradigms increases our knowledge of dynamical inter-brain mechanisms (Wilms et al., 2010).

Few fMRI studies have also focused on the influence of interpersonal coupling on information transmission during verbal (Stephens et al., 2010) or non-verbal communication (Schippers et al., 2010). Whereas these two experiments involved an offline unidirectional interaction, designed tasks were rather ecological and engaging. Schippers et al. (2010) introduced the social game of charades in neuroimaging research as a motivating task to study gestural communication in romantic couples. Individual data suggested that such communication relies on a combination of simulation and, during decoding, mentalizing brain structures such as the ventromedial prefrontal cortex. Stephens et al. (2010) captured brain activity from both speakers and listener when telling a real-life story. On average, during successful communication the listener's brain responses mirrored the speaker's brain responses with some temporal delays. Using advanced connectivity measures, these two studies revealed temporal and spatial coupling between communicating brains, which was interpreted as a putative mechanism by which brains convey information.

## Interpersonal coupling: a nascent basis for social cognition?

To uncover the neural underpinning of our abilities to understand each other's intentions and feelings remains one of the main objectives in social neuroscience. Recent integrative proposals have emphasized a collaborative role of the putative mirror neuron system and the mentalizing network, which may both be recruited during our highly complex social life (Keysers and Gazzola, 2007). Surely the introduction of truly interactive neuro-imaging paradigms will shed new light on social cognition, in complement to conventional observational paradigms.

In line with the majority of current cognitivist and individualistic perspectives on social understanding, one may consider social processes depicted in our taxonomy solely as internalized mechanisms implemented in specific brain modules. However, such a reductionism could conceal potential *non-local mechanisms* that could occur *across* interacting people. In this regard, bringing online and reciprocal social interaction into experimental paradigms is also a step for the exploration of interpersonal coupling. In turn, interpersonal coupling may play a fundamental role in most of the social cognitive processes mentioned in this article. Research on these non-local emergent mechanisms—also referred to as interactive alignment, resonance, phase synchronization, etc.—is becoming an increasingly influential movement, as illustrated by recent hypotheses in neuroscience (Hasson et al., 2012), philosophy (Di Paolo and De Jaegher, 2012) and by nearly all findings presented throughout the present review.

## Future directions

In this short review paper, we have provided a comprehensive taxonomy of mutually related but mostly independent research works in neuroscience of social interaction, situating them in the frame of general organization principles of social cognition. It is clear that research on the substrates of social interaction is entangled with the study of non-local mechanisms in place during mutual give and take. We still know so little about interpersonal coupling. Does it play a causal role in initiating and maintaining action coordination (Lindenberger et al., 2009)? Does it provide a natural basis for communication and creation of a shared world (Hasson et al., 2012)? How is it related to the various social (implicit and/or altruistic) processes at play during social interaction? And finally, does it play a role in the creation and sustaining of intense pairbonded relationships, a special feature of anthropoid primate life (Dunbar and Shultz, 2007)?

We see several paths for future research. To date no hyperscanning experiment has compared interpersonal dynamics across populations with normal vs. impaired social abilities. If coupling between individuals does play a role for successful social interaction, its investigation is certainly of clinical relevance. Future studies digging into the characterization of social disorders from an interpersonal point of view might benefit from recent technological advances in the monitoring of brain function during realistic social interactions (see e.g., Suda et al., 2011). Furthermore, neural mechanisms subtending social interaction need to be studied systematically across species (Frith and Frith, 2012). So far no comparative study investigated the joint dynamics of brain activities across non-humans. Finally, in future research it might be of interest to look at possible relations between psycho-pharmacological factors and interpersonal couplings. For instance, is there an influence of neuro-hormones oxytocin or vasopressin on how we synchronize with others?

In conclusion, further probing into inter-brain mechanisms at play during natural social interaction seems to be a great challenge ahead. This can only be answered outside the boundaries of isolated bodies and minds.

### Conflict of interest statement

The authors declare that the research was conducted in the absence of any commercial or financial relationships that could be construed as a potential conflict of interest.
